# A conditional Pax6 depletion study with no morphological effect on the adult mouse corneal epithelium

**DOI:** 10.1186/s13104-018-3812-9

**Published:** 2018-10-05

**Authors:** Natalie J. Dorà, Martine Manuel, Dirk-Jan Kleinjan, David J. Price, J. Martin Collinson, Robert E. Hill, John D. West

**Affiliations:** 1 Centre for Integrative Physiology, Biomedical Sciences, University of Edinburgh Medical School, Hugh Robson Building, George Square, Edinburgh, EH8 9XD UK; 2 Centre for Discovery Brain Sciences, Biomedical Sciences, University of Edinburgh Medical School, Hugh Robson Building, George Square, Edinburgh, EH8 9XD UK; 3 Medical and Developmental Genetics Section, MRC Human Genetics Unit, MRC IGMM, University of Edinburgh, Western General Hospital, Crewe Road, Edinburgh, EH4 2XU UK; 4 School of Medicine, Medical Sciences and Nutrition, Institute of Medical Sciences, University of Aberdeen, Foresterhill, Aberdeen, AB25 2ZD UK; 5 Centre for Integrative Physiology, Clinical Sciences, University of Edinburgh Medical School, Hugh Robson Building, George Square, Edinburgh, EH8 9XD UK; 6 Present Address: Biology Teaching Organisation, University of Edinburgh, Ashworth Laboratories, Charlotte Auerbach Road, King’s Buildings, Edinburgh, EH9 3FL UK; 7 Present Address: Centre for Mammalian Synthetic Biology, University of Edinburgh, Roger Land Building, Alexander Crum Brown Road, King’s Buildings, Edinburgh, EH9 3FF UK

**Keywords:** Cornea, Corneal epithelium, Cre-*loxP*, CAG-CreER, *Krt19*-CreER, Pax6, Mouse, Mosaic transgene expression

## Abstract

**Objective:**

The corneas of heterozygous *Pax6*^+*/*−^ mice develop abnormally and deteriorate further after birth but it is not known whether the postnatal deterioration is predetermined by abnormal development. Our objective was to identify whether depletion of Pax6 in adult mice caused any corneal abnormalities, similar to those in *Pax6*^+*/*−^ mice, where Pax6 levels are low throughout development and adulthood. We used two tamoxifen-inducible, Cre-*loxP* experimental strategies to deplete Pax6 either ubiquitously or in a restricted range of cell types.

**Results:**

In a preliminary study, ubiquitous depletion of Pax6 by tamoxifen treatment of E9.5 CAG-CreER^*Tg/*−^;*Pax6*^*fl/fl*^ embryos affected eye development. Tamoxifen treatment of 12-week old, adult CAG-CreER^*Tg/*−^;*Pax6*^*fl/*+^ and CAG-CreER^*Tg/*−^;*Pax6*^*fl/fl*^ mice resulted in weak and/or patchy Pax6 immunostaining in the corneal epithelium but caused no corneal abnormalities. GFP staining in tamoxifen-treated CAG-CreER^*Tg/*−^;RCE:*loxP* reporter mice was also patchy. We attribute patchy Pax6 staining to mosaic deletion of the *Pax6*^*fl*^ allele, probably caused by mosaic CAG-CreER^*Tg*^ expression. In a parallel study, we treated adult *Krt19*-CreER^*Tg/*−^;*Pax6*^*fl/*+^ mice with tamoxifen to try to deplete Pax6 in limbal epithelial stem cells (LESCs) which replenish the corneal epithelium. However, Pax6 staining remained strong after a 12-week chase period so the *Krt19*-CreER^*Tg/*−^ transgene may have failed to target LESCs.

**Electronic supplementary material:**

The online version of this article (10.1186/s13104-018-3812-9) contains supplementary material, which is available to authorized users.

## Introduction

The mouse cornea comprises an outer epithelium of 5–6 cell layers, a thick stroma and an inner endothelium. The corneal epithelium is maintained by limbal epithelial stem cells (LESCs), in the ring-shaped limbus, which is a transition zone between the corneal epithelium and the conjunctiva. The LESCs replace themselves and produce transient (or transit) amplifying cells (TACs) that move centripetally across the basal corneal epithelium and produce more differentiated daughter cells, which move apically, through the epithelial layers, and are shed from the surface [1].

The *Pax6* gene, encoding the Pax6 transcription factor, is expressed in the brain, pancreas, olfactory system and several eye tissues, including the corneal and limbal epithelia, and is critical for eye development [2]. Low levels of Pax6 throughout development of heterozygous *Pax6*^+*/*−^ mice cause small eyes, aniridia plus lens and corneal defects [3–8]. The newborn *Pax6*^+*/*−^ cornea has a thin epithelium and the adult cornea deteriorates further, because the epithelium is poorly maintained and limbal blood vessels invade the stroma [6–8]. The adult *Pax6*^+*/*−^ corneal epithelium is thin and fragile, cell turnover is elevated, centripetal movement is disrupted and goblet cells accumulate. The expression of keratin 12 (K12), which is regulated by Pax6 [9], is delayed and immunostaining is weak and patchy [6–8, 10, 11]. Indirect evidence suggests that reduced Pax6 causes LESC deficiency in both *PAX6*^+*/*−^ humans and *Pax6*^+*/*−^ mice and this may underlie adult corneal deterioration [1, 12].

It is not known if all corneal abnormalities in adult *Pax6*^+*/*−^ mice are predetermined by abnormal development or whether some are caused by reduced Pax6 in the adult. Our aim was to determine whether depletion of Pax6 in adult mice caused corneal abnormalities, comparable to those reported for *Pax6*^+*/*−^ mice. We used one experimental strategy to deplete Pax6 ubiquitously and another to deplete Pax6 in LESCs.

## Main text

### Materials and methods

#### Mice

To deplete Pax6 ubiquitously, CAG-CreER^*Tg/*−^;*Pax6*^*fl/*+^ mice were produced by crossing hemizygous CAG-CreER^*Tg/*−^ mice (formal transgene name: Tg(CAG-cre/Esr1*)5Amc) [13] to heterozygous *Pax6*^*fl/*+^ mice (formal name: *Pax6*^*tm1Ued/*+^) [14]. Superscript symbols ‘*Tg/*−’ and ‘−*/*−’ are used to distinguish hemizygous CAG-CreER^*Tg/*−^ mice and non-transgenic CAG-CreER^−*/*−^ siblings.

Keratin 19 (*Krt19* gene; K19 protein) is expressed in the basal epithelium of the mouse limbus and conjunctiva but not the cornea [15]. To try to target LESCs in the limbal epithelium, *Krt19*-CreER^*Tg/*−^;*Pax6*^*fl/*+^ mice were produced by crossing hemizygous *Krt19*-CreER^*Tg/*−^ mice (formal transgene name: *Krt19*^*tm1(cre/ERT)Ggu*^) [16] to heterozygous *Pax6*^*fl/*+^ mice.

RCE:*loxP* mice have the R26R CAG-boosted EGFP (RCE) reporter allele with an upstream *loxP*-flanked STOP cassette (formal transgene name: *Gt(ROSA)26Sor*^*tm1.1(CAG*−*EGFP)Fsh*^) [17]. CAG-CreER^*Tg/*−^;RCE:*loxP* mice with tamoxifen-inducible expression of GFP were bred by crossing CAG-CreER^*Tg/*−^ and RCE:*loxP* mice. *Krt19*-CreER^*Tg/*−^;RCE:*loxP* mice were bred by crossing *Krt19*-CreER^*Tg/*−^ and RCE:*loxP* mice.

Mice were maintained on a predominantly CBA/Ca genetic background and genotyped by PCR [13, 14, 16]. Some additional samples from mice on a CD-1 or (C57BL/6 × CBA/Ca)F1 genetic background from other studies [18, 19], were also analysed.

To activate CreER in adult mice, tamoxifen (Sigma-Aldrich) was freshly prepared in corn oil (25–40 mg/ml) by sonication in a 40 °C water bath and adjusted to 100 μg/g body weight in 0.1 ml. Mice of both sexes were injected intraperitoneally with tamoxifen at 12 weeks on 5 consecutive days and analysed 3 days later (no chase group) or after chase periods of 6 or 12 weeks. Control mice were injected with 0.1 ml corn oil. Mice were culled by cervical dislocation, following overdose of gaseous halothane, and eyes were enucleated. Procedures for the induction of Cre expression in embryos at embryonic day (E) 9.5 and the subsequent collection of E13.5 fetal samples are described elsewhere [19]. Tamoxifen treatment causes CreER to move to the nucleus and recombine *loxP* sites to convert the functional *Pax6*^*fl*^ floxed allele to a *Pax6*^*Δ*^ null allele or express the GFP lineage marker in the target cells and their progeny. This should occur in all cell types in CAG-CreER^*Tg/*−^ mice, because CreER is expressed ubiquitously from the CAG promoter, but only in specific cell types in *Krt19*-CreER^*Tg/*−^ mice.

#### Analysis

Tissue samples were fixed in 4% paraformaldehyde overnight at 4 °C. Fetal heads were processed to OCT compound and stored frozen before cryosections were cut and stained with haematoxylin and eosin (H&E) [19, 20]. Adult eyes were processed to paraffin wax, then 7 μm sections were cut, mounted on glass slides and stained with H&E or periodic acid-Schiff (PAS) stain [20]. Stained sections were photographed and measured using a Zeiss Axioplan-2 microscope and calibrated Zeiss Axiovision 4.8 digital camera system. Numerical data are included in Additional file 1 and measurements were compared by Student’s t-test.

Immunohistochemistry methods are described elsewhere [20]. Briefly, wax sections, mounted on glass slides were heat-treated to unmask antigens, then incubated with blocking serum, followed by primary antibody, biotinylated secondary antibody, avidin–biotin reagent and 3,3′-diaminobenzidine (DAB) stain. Sections were then lightly counterstained with haematoxylin, dehydrated and slides were mounted with DPX mounting medium under coverslips. Negative control slides were treated with blocking serum instead of primary antibody. The antibodies used for Pax6 and K12 immunostaining were as described elsewhere [20], except that the secondary antibody was biotinylated rabbit anti-mouse, diluted 1:200 (E0433 from Dako, Ely, UK). For GFP immunostaining, the primary antibody was rabbit anti-GFP diluted 1:500 (ab290 from Abcam, Cambridge, UK) and the secondary antibody was biotinylated goat anti-rabbit, diluted 1:200 (Sc-2012 from SantaCruz Biotechnology, Heidelberg, Germany).

### Results

#### Ubiquitous depletion of Pax6 in embryos

For another study, CAG-CreER^*Tg/*−^;*Pax6*^*fl/fl*^ and CAG-CreER^*Tg/*−^;*Pax6*^*fl/*+^ embryos were exposed to tamoxifen at E9.5 and culled at E13.5 [19]. By E13.5 tamoxifen-treated CAG-CreER^*Tg/*−^;*Pax6*^*fl/fl*^ fetuses (with two floxed *Pax6*^*fl*^ alleles) had smaller eyes and lenses than CAG-CreER^*Tg/*−^;*Pax6*^*fl/*+^ fetuses (Additional file 1 and Additional file 2: Fig. S1). This showed that tamoxifen-mediated depletion of Pax6 could affect eye development.

#### Ubiquitous depletion of Pax6 in adults

The preliminary result with fetal eyes encouraged us to investigate whether tamoxifen-mediated, depletion of Pax6 in adults caused any corneal abnormalities, similar to those in *Pax6*^+*/*−^ mice, where Pax6 is low throughout development. Pax6-depletion in adults is unlikely to reproduce *Pax6*^+*/*−^ developmental defects but adult corneal deterioration could be mediated via Pax6-deficiency in adult LESCs, the LESC niche, the corneal epithelium or other ocular tissues [5, 10]. We compared the effects of tamoxifen treatment of CAG-CreER^*Tg/*−^;*Pax6*^*fl/*+^ mice to several genotype and treatment controls, which were included to control for any unexpected effects of the CAG-CreER transgene or the floxed *Pax6*^*fl*^ allele alone [20–24].

**Fig. 1:**
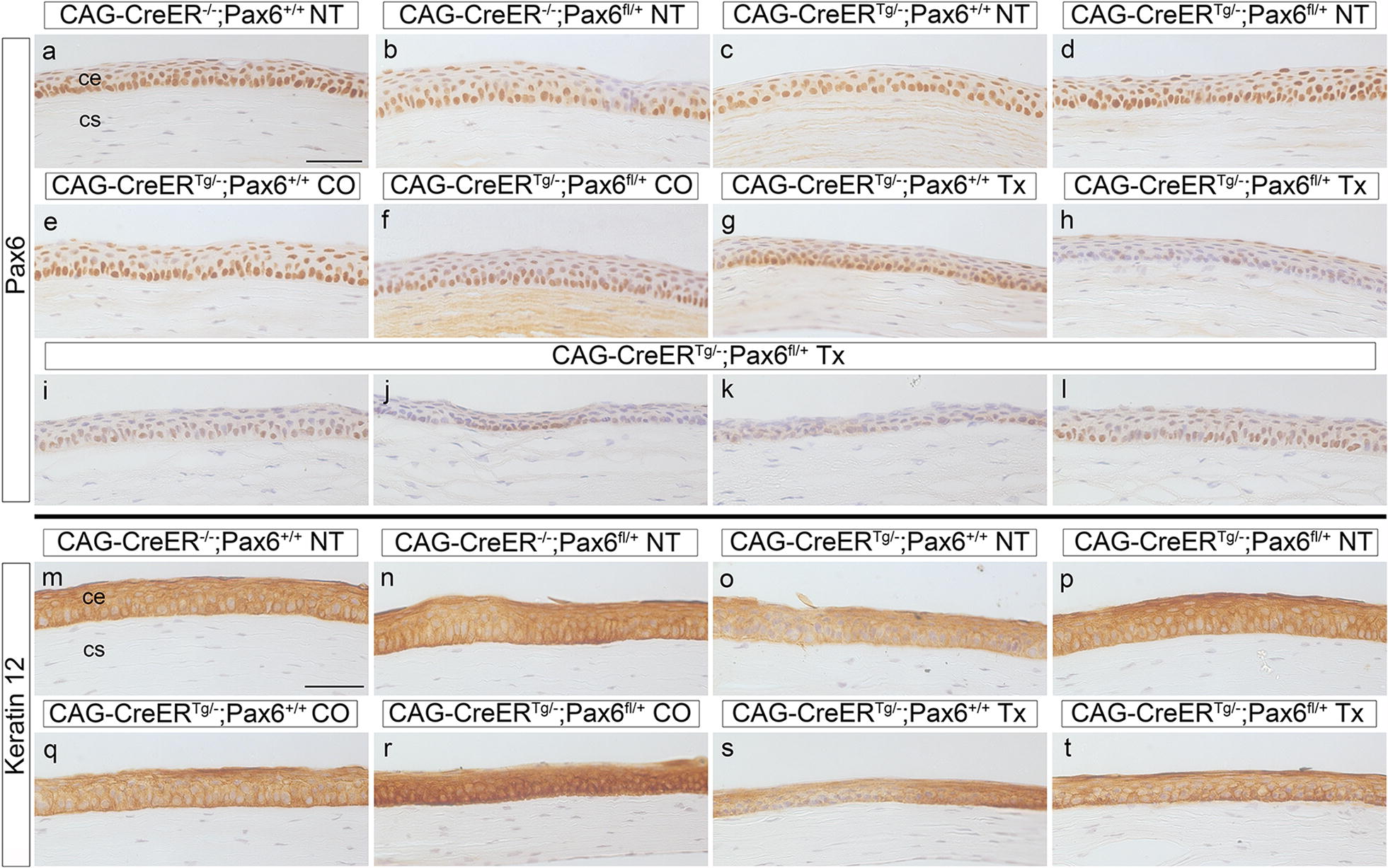
Pax6 and keratin 12 immunohistochemistry of corneal epithelium after a 6-week chase period. **a**–**l** Central region of the adult corneal epithelium, immunostained for Pax6 (brown DAB endpoint) and counterstained with haematoxylin, showing various controls with different genotype and treatment combinations, 6 weeks after treatment (**a**–**g**), and several examples of weak and/or patchy staining of CAG-CreER^Tg/−^;*Pax6*^*fl/*+^corneas, 6 weeks after treating with tamoxifen (**h**–**l**). **m**–**t** Central region of the adult corneal epithelium, immunostained for keratin 12 (brown DAB endpoint) and counterstained with haematoxylin, showing various controls with different genotype and treatment combinations, 6 weeks after treatment (**m**–**s**), and a CAG-CreER^Tg/−^;*Pax6*^*fl/*+^ cornea, 6 weeks after treating with tamoxifen (**t**). Scale bars = 50 μm. *ce* corneal epithelium, *CO* corn oil treatment, *cs* corneal stroma, *NT* no treatment, *Tx* tamoxifen treatment. All mice were on a predominantly CBA/Ca genetic background

**Fig. 2:**
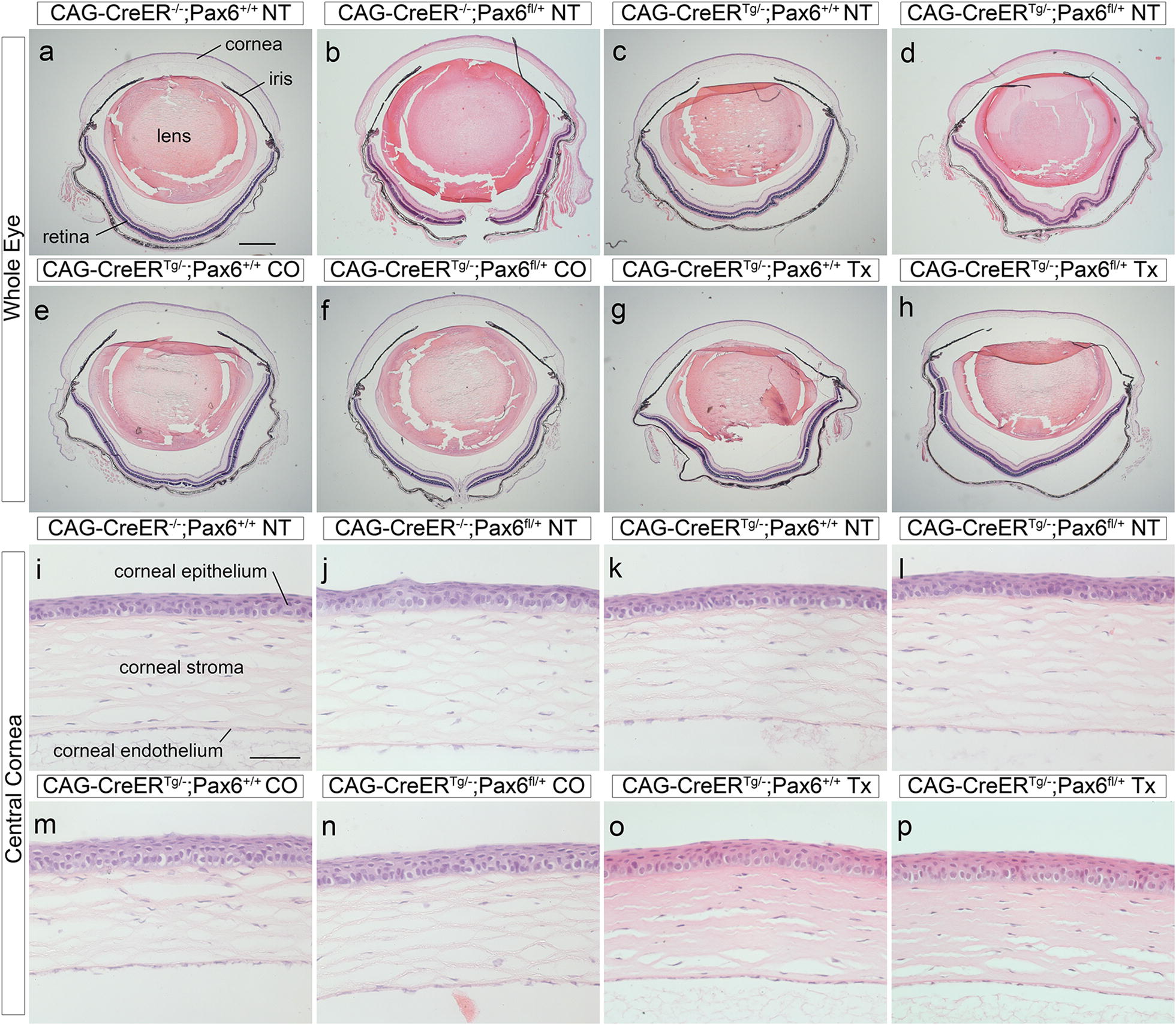
Histology of whole eyes and corneas after a 6-week chase period. **a**–**h** Whole eye morphology in H&E stained sections showing grossly normal eye morphology in all the controls (**a**–**g**) and in CAG-CreER^Tg/−^;*Pax6*^*fl/*+^ eyes, 6 weeks after tamoxifen treatment (**h**). **i**–**p** H&E stained central corneas showing normal corneal morphology in all the controls (**i**–**o**) and in CAG-CreER^Tg/−^;*Pax6*^*fl/*+^ corneas, 6 weeks after tamoxifen treatment (**p**). Scale bars: **a** (for **a**–**h**) = 500 μm; **i** (for **i**–**p**) = 50 μm. *CO* corn oil treatment, *NT* no treatment, *Tx* tamoxifen treatment

**Fig. 3:**
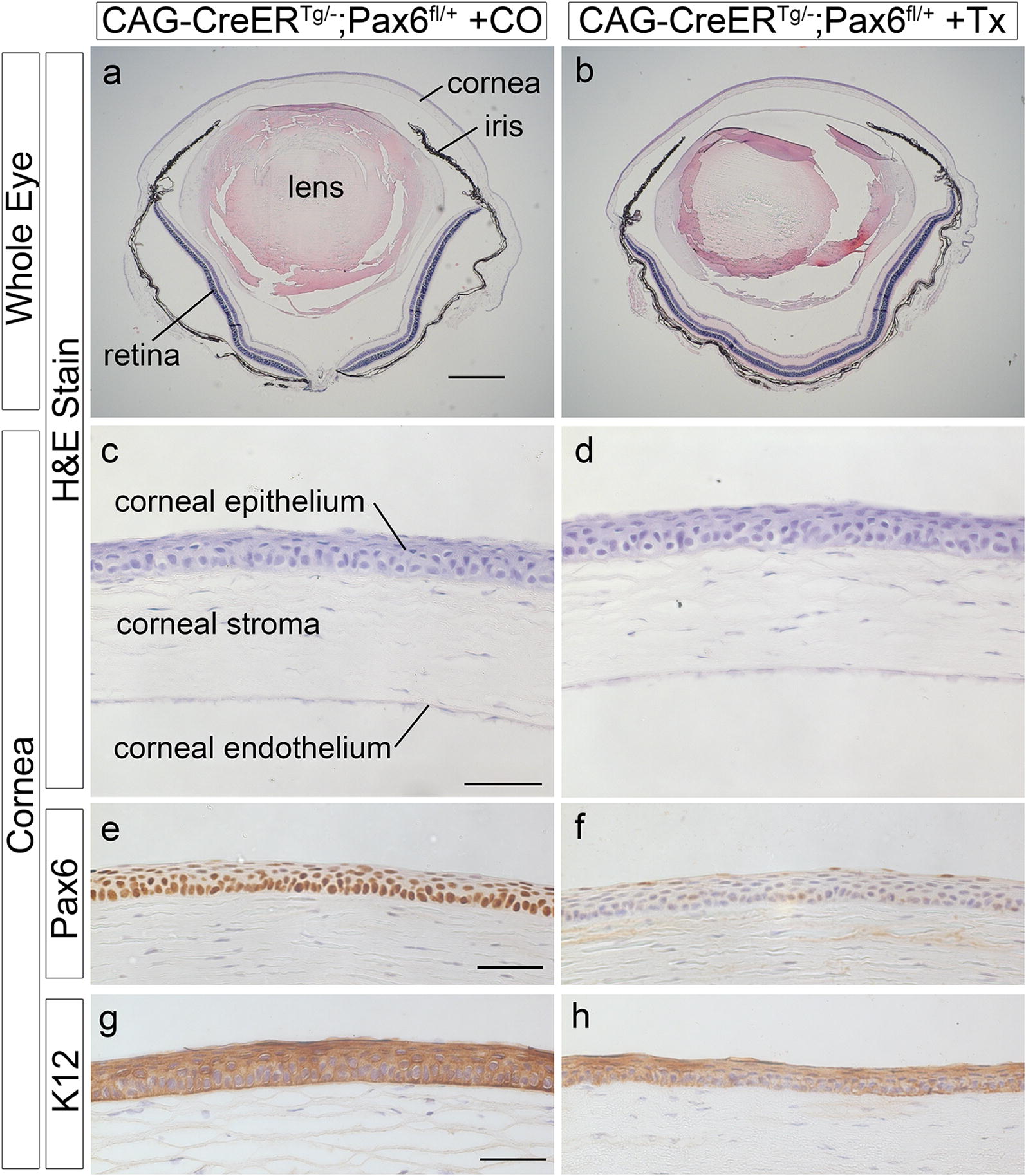
Histology and immunohistochemistry of CAG-CreER^Tg/−^;*Pax6*^*fl/*+^ tissues after a 12 week chase period. **a**–**d** H&E stained sections of whole eyes (**a**, **b**) and central cornea (**c**, **d**) of corn oil treated controls and tamoxifen treated CAG-CreER^Tg/−^;*Pax6*^*fl/*+^ mice, 12 weeks after treatment. **e**–**h** Immunostained sections (brown DAB endpoint) for Pax6 (**e**, **f**) and keratin 12 (**g**, **h**) 12 weeks after treatment. Scale bars: **a** (for **a**, **b**) = 500 μm; **c** (for **c**, **d**), **e** (for **e**, **f**) and **g** (for **g**, **h**) = 50 µm. *CO* corn oil treatment, *Tx* tamoxifen treatment

Following tamoxifen treatment at 12 weeks and a 6-week chase, Pax6 immunostaining was positive in the corneal epithelia of all the control combinations (Fig. 1a–g). Although immunohistochemistry was not quantified, Pax6 appeared to be weak and/or patchy in the corneal epithelia of CAG-CreER^*Tg/*−^;*Pax6*^*fl/*+^ mice, treated with tamoxifen as adults (Fig. 1h–l). However, eye and corneal morphology appeared grossly normal (apart from some processing artefacts), with no blood vessels visible in the cornea (Fig. 2). PAS-positive goblet cells were not detected in the corneal epithelium (data not shown) and there was little or no effect on K12 staining (Fig. 1m–t). Similar results were obtained after a 12-week chase and, again, corneal morphology appeared grossly normal (Fig. 3). For comparison, previously published corneal histology and immunostaining are shown for wild-type *Pax6*^+*/*+^ and heterozygous *Pax6*^+*/*−^ eyes in Additional file 2: Fig. S2. GFP immunostaining of eyes from tamoxifen-treated CAG-CreER^*Tg/*−^;RCE:*loxP* reporter mice showed mosaic expression in the corneal epithelium (Additional file 2: Fig. S3).

We did not include CAG-CreER^*Tg/*−^;*Pax6*^*fl/fl*^ mice, with two floxed *Pax6*^*fl*^ alleles, in the main study because severe, global depletion of Pax6 in these mice results in diabetes [18]. However, Pax6 immunostaining of corneas from CAG-CreER^*Tg/*−^;*Pax6*^*fl/fl*^ mice, produced for another study [18], showed that Pax6 protein was not eliminated following tamoxifen treatment and a 6-week chase period (Additional file 2: Fig. S4).

#### Targeting Pax6-depletion to LESCs

To try to deplete *Pax6* in LESCs we treated *Krt19*-CreER^*Tg/*−^;*Pax6*^*fl/*+^ mice with tamoxifen at 12 weeks and analysed the results after a 12-week chase period, to allow sufficient time for treated LESCs to replenish the corneal epithelium. Pax6 and K12 staining appeared normal and no corneal morphological abnormalities were seen (Additional file 2: Fig. S5). Tamoxifen-treated *Krt19*-CreER^*Tg/*−^;RCE:*loxP* reporter mice produced patchy GFP reporter immunostaining in the conjunctiva, some sparse staining in the limbus but no staining in the corneal epithelium (Additional file 2: Fig. S6).

### Discussion

Tamoxifen treatment to deplete Pax6 in E9.5 CAG-CreER^*Tg/*−^;*Pax6*^*fl/fl*^ embryos affected eye morphology by E13.5 but did not prevent lens development. The lens placode forms around E9.5 and is absent in *Pax6*^−*/*−^ homozygotes [25], so E9.5 tamoxifen-treatment was probably too late to prevent lens development in the CAG-CreER^*Tg/*−^;*Pax6*^*fl/fl*^ embryos but we did not investigate whether any Pax6 remained at E13.5.

As tamoxifen treatment of *Krt19*-CreER^*Tg/*−^;RCE:*loxP* mice did not produce any GFP-positive corneal epithelial cells after a 12-week chase, it is likely that this strategy failed to target LESCs. With hindsight, the *Krt14*-CreER^*Tg/*−^ mouse may have been more suitable for targeting expression to LESCs, as this has been successful in lineage tracing experiments [26, 27].

The normal corneal morphology of the tamoxifen-treated adult CAG-CreER^*Tg/*−^;*Pax6*^*fl/*+^ mice is consistent with the possibility that normal Pax6 levels are not required in the adult to maintain the corneal epithelium. This would suggest that deterioration of the adult *Pax6*^+*/*−^ corneal epithelium was predetermined during development. However, we also need to consider technical explanations for our results, particularly as corneal effects of Pax6-deficiency have now been corrected successfully in adult mice [28, 29].

One possibility is that our investigation was undermined by mosaic deletion of the *Pax6*^*fl*^ allele. The presence of some Pax6-positive cells in the corneal epithelia of tamoxifen-treated CAG-CreER^*Tg/*−^;*Pax6*^*fl/fl*^ mice suggests that mosaicism occurred in these mice as well as CAG-CreER^*Tg/*−^;RCE:*loxP* reporter mice. We, therefore, suggest that mosaic *Pax6*^*fl*^ deletion was caused by mosaic CAG-CreER^*Tg*^ transgene expression but recombination of *Pax6*^*fl*^ *loxP* sites could also be inefficient for other reasons. Mosaic *Pax6*^*fl*^ deletion would lead to a mixture of *Pax6*^*Δ/Δ*^, *Pax6*^*fl/Δ*^ and *Pax6*^*fl/fl*^ cells in CAG-CreER^*Tg/*−^;*Pax6*^*fl/fl*^ mice or a mixture of *Pax6*^*Δ/*+^ and *Pax6*^*fl/*+^ cells in CAG-CreER^*Tg/*−^;*Pax6*^*fl/*+^ mice.

The effects of mixtures of wild-type and *Pax6*^−*/*−^ or *Pax6*^+*/*−^ cells, in ocular tissues, have been investigated using mouse chimaeras. Eye development was abnormal in *Pax6*^+*/*+^↔*Pax6*^−*/*−^ chimaeras [30–33] but *Pax6*^+*/*+^↔*Pax6*^+*/*−^ chimaeras had normal eyes [5, 10, 33] with normal corneal morphology [10]. It was suggested that unknown signals from wild-type cells in the cornea and/or other ocular tissues might rescue the *Pax6*^+*/*−^ cells [10]. This might also occur in tamoxifen-treated CAG-CreER^*Tg/*−^;*Pax6*^*fl/*+^ corneas if the conditional *Pax6*^*fl*^ allele is not deleted in all cells.

Although the corneal epithelia of tamoxifen-treated CAG-CreER^*Tg/*−^;*Pax6*^*fl/fl*^ mice contained many Pax6-positive cells (Additional file 2: Fig. S4), no Pax6 protein was detected by immunofluorescence in most pancreatic islet cells in equivalent mice from the same study [18]. Apparent differences in frequencies of Pax6-positive cells between these two tissues may reflect genuine biological differences rather than technical differences in detecting Pax6-positive cells. Mosaic CAG-CreER^*Tg*^ expression might be more common and/or recombination of *loxP* sites less efficient in the corneal epithelium than pancreatic islets, resulting in mosaic *Pax6*^*fl*^ deletion in the cornea.

Mosaic reporter expression also occurred in the corneal epithelium of CAG-CreER;R26R-*LacZ* and CAG-CreER;R26R-mT/mG reporter mice in a lineage-tracing experiment [34]. This lineage tracing experiment was undertaken after the present study and, with hindsight, it would be worth investigating whether mosaic transgene expression is more common for specific tissues and/or specific CAG-CreER;*loxP* combinations.

## Limitations

We evaluated corneal histology, the absence of goblet cells and K12 immunohistochemistry. Future investigations could include additional endpoints and markers.

In lineage-tracing experiments, labelled cells produced by tamoxifen-treated LESCs took at least 14 weeks to replace the whole corneal epithelium [26, 27, 34]. Thus, although our chase time of 12 weeks should have identified corneal defects attributable to Pax6-depletion in LESCs or the niche, it might not have been sufficient to produce the maximum effects.

We did not investigate how effectively Pax6 protein was depleted in other CAG-CreER^*Tg/*−^;*Pax6*^*fl/*+^ ocular tissues or whether mosaic transgene expression occurred in those tissues. Also, DNA or RNA methods were not used to confirm that at least some floxed *Pax6*^*fl*^ alleles were converted to *Pax6*^*Δ*^ in the corneal epithelium.

## Additional files

**Additional file 1.** Fetal eye measurements.

**Additional file 2: Fig S1.** E13.5 fetal eye morphology following tamoxifen treatment at E9.5. **Fig. S2.** Previously published histology and immunohistochemistry of adult wild-type and heterozygous *Pax6*^*+/−*^ mouse eyes. **Fig. S3.** Expression of GFP reporter in corneal epithelium of CAG-CreER^*Tg/−*^; RCE:*loxP* mice after tamoxifen treatment and different chase periods. **Fig. S4.** Pax6 immunohistochemistry of CAG-CreER^Tg/*−*^;*Pax6*^*fl/+*^ and CAG-CreER^Tg/*−*^;*Pax6*^*fl/fl*^ corneal epithelia after a 6-week chase period. **Fig. S5.** Histology and immunohistochemistry of *Krt19*-CreER^Tg/*−*^;Pa*x6*^*fl/+*^ tissues after a 12 week chase period. **Fig. S6.** Expression of GFP reporter in corneal epithelium of *Krt19*-CreER^*Tg/−*^; RCE:*loxP* mice after tamoxifen treatment and different chase periods.

## Abbreviations

DAB: 3,3′-diaminobenzidine
DPX: distyrene plasticizer xylene
E: embryonic day
EGFP: enhanced green fluorescent protein
GFP: green fluorescent protein
H&E: haematoxylin and eosin
K12: keratin 12
K19: keratin 19
LESC: limbal epithelial stem cell
OCT compound: optimal cutting temperature compound
PAS: periodic acid-Schiff
TAC: transient (or transit) amplifying cell
