# Supplementary material for: A conditional Pax6 depletion study with no morphological effect on the adult mouse corneal epithelium
Source: BMC Res Notes. 2018 Oct 5;11:705. doi: 10.1186/s13104-018-3812-9 (PMC6173925; doi:10.1186/s13104-018-3812-9)
Supplement: Supplementary file 2 — Additional file 2: Fig S1. E13.5 fetal eye morphology following tamoxifen treatment at E9.5. Fig. S2. Previously published histology and immunohistochemistry of adult wild-type and heterozygous Pax6+/− mouse eyes. Fig. S3. Expression of GFP reporter in corneal epithelium of CAG-CreERTg/−; RCE:loxP mice after tamoxifen treatment and different chase periods. Fig. S4. Pax6 immunohistochemistry of CAG-CreERTg/−;Pax6fl/+ and CAG-CreERTg/−;Pax6fl/fl corneal epithelia after a 6-week chase period. Fig. S5. Histology and immunohistochemistry of Krt19-CreERTg/−;Pax6fl/+ tissues after a 12 week chase period. Fig. S6. Expression of GFP reporter in corneal epithelium of Krt19-CreERTg/−; RCE:loxP mice after tamoxifen treatment and different chase periods. [file 13104_2018_3812_MOESM2_ESM.pdf]

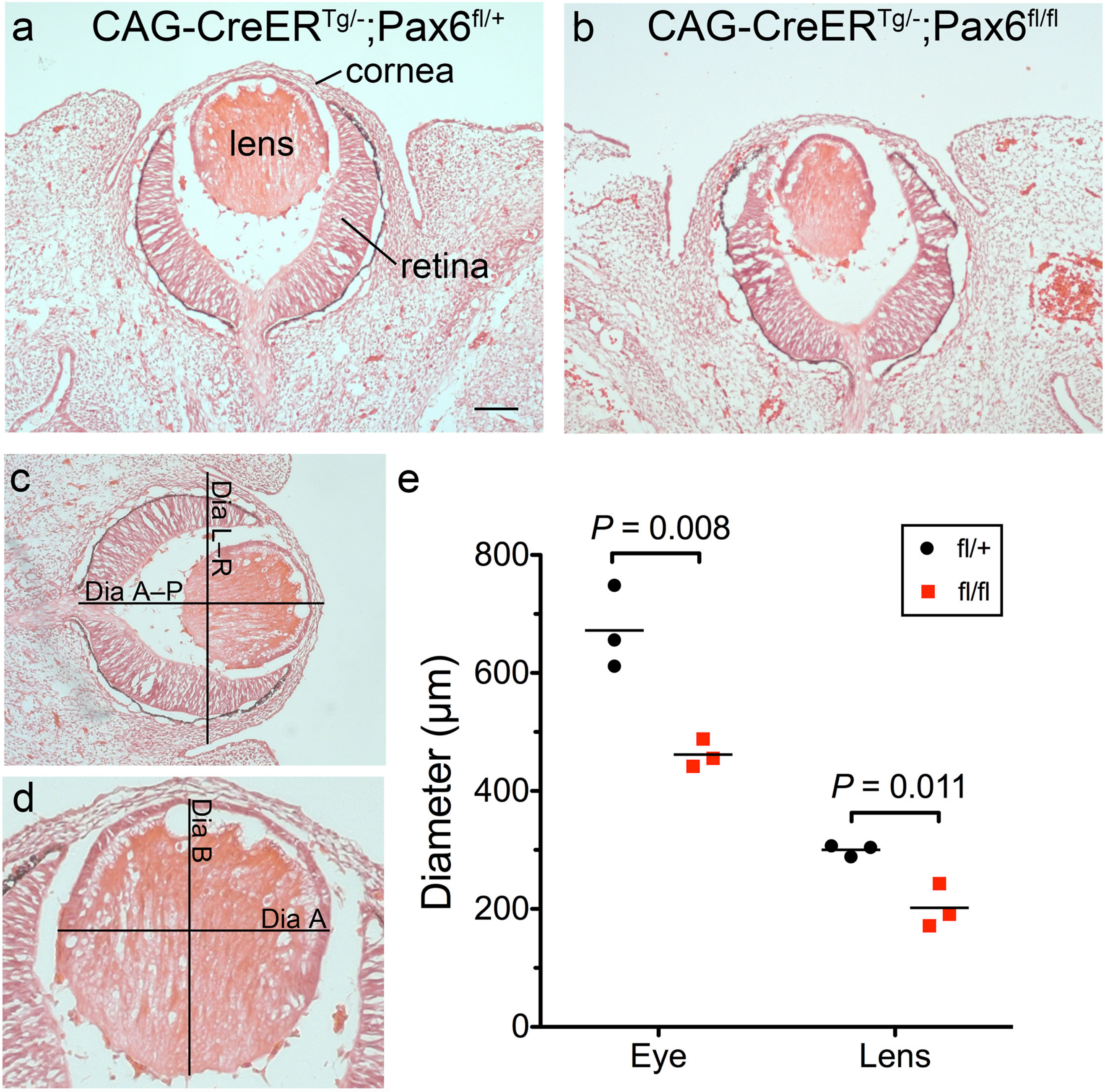

Additional file 2: Fig. S1. E13.5 fetal eye morphology following tamoxifen treatment at E9.5. **(a, b)** H&E staining of cryosections of eyes from E13.5 CAG-CreER$^{Tg/-}$;*Pax6*$^{fl/+}$ (a) and CAG-CreER$^{Tg/-}$;*Pax6*$^{fl/fl}$ (b) fetuses, after treating pregnant females with tamoxifen at E9.5. **(c, d)** Orthogonal measurements of fetal eye (c) and lens (d) diameters. **(e)** Comparisons of CAG-CreER$^{Tg/-}$;*Pax6*$^{fl/+}$ (fl/+) and CAG-CreER$^{Tg/-}$;*Pax6*$^{fl/fl}$ (fl/fl) eye and lens diameters. The diameter values shown are means for left and right eyes or lenses, which were each calculated as the mean of two orthogonal measurements, as shown in (c) and (d). The individual diameters are shown for three fetuses of each genotype and the group means are shown as horizontal bars. Different genotypes were compared by Student's *t*-test and *P*-values are shown in the figure. Although there were only 3 fetuses in each group, there was a clear trend for CAG-CreER$^{Tg/-}$;*Pax6*$^{fl/fl}$ fetuses to have smaller eyes and lenses with no overlap between genotypes. Scale bar in (a) for (a, b) = 100 µm. Abbreviations: A–P, anterior–posterior; Dia, diameter; L–R, left–right. Mice were on a predominantly (C57BL/6 ×CBA/Ca)F1 genetic background and were produced for another study [19].

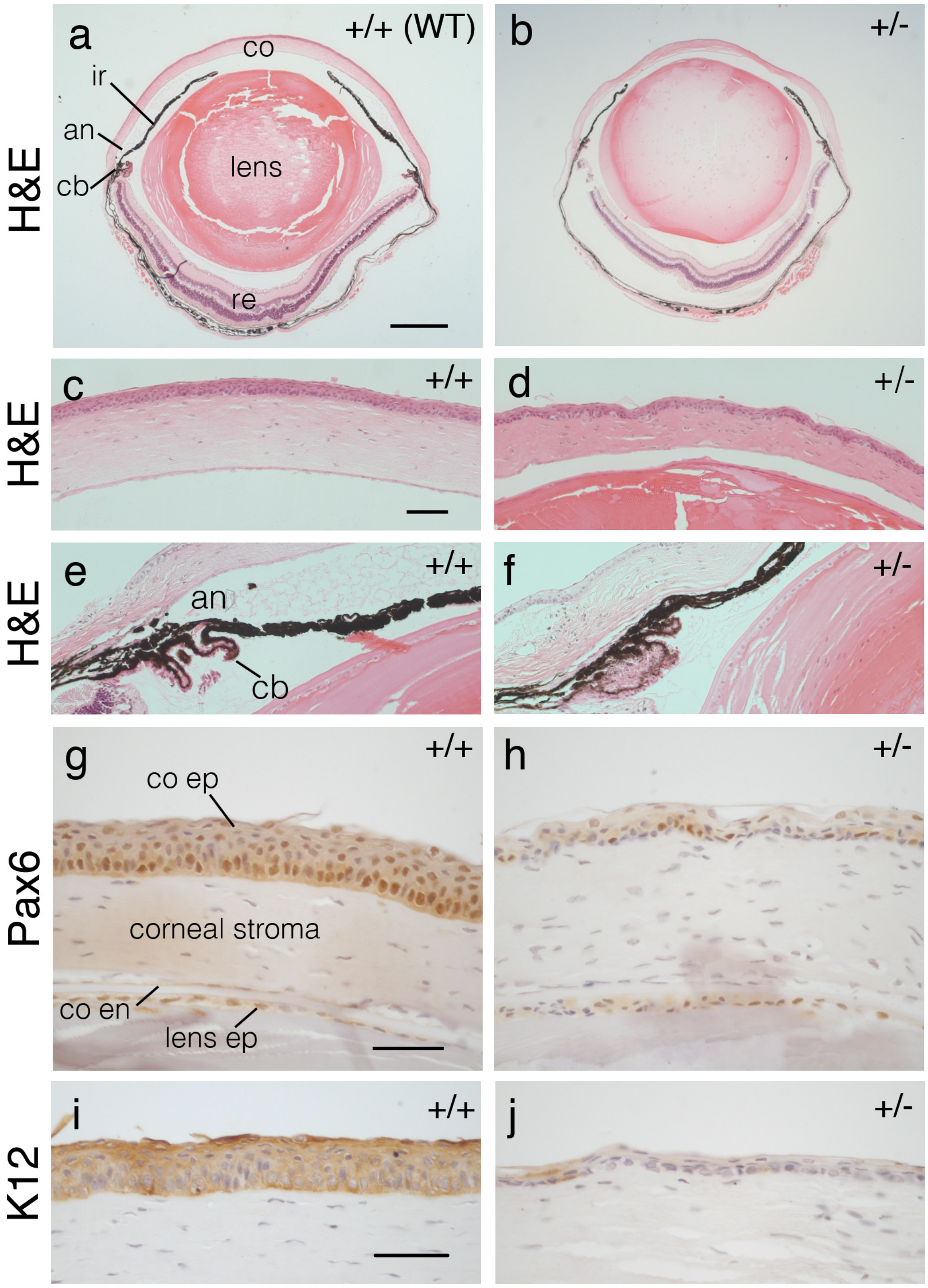

Additional file 2: Fig. S2. Previously published histology and immunohistochemistry of adult wild-type and heterozygous *Pax6*$^{+/-}$ mouse eyes.

(a–f) H&E stained sections of eyes from 12-week old wild-type (WT), *Pax6*$^{+/+}$ (+/+) (a, c, e) and heterozygous *Pax6*$^{+/-}$ (+/-) (b, d, f) mice to show morphology of the whole eye (a, b), the central cornea (c, d) and the ciliary body plus irido-corneal angle (e, f). **(g–j)** Immunostained sections (brown DAB endpoint) of wild-type (g, i) and heterozygous *Pax6*$^{+/-}$ (h, j) eyes to show corneal morphology and staining for Pax6 (g, h) and K12 (i, j). Scale bars: a (for a, b) = 500 µm; c (for c–f), g (for g, h) and i (for i, j) = 50 µm. Abbreviations: an, irido-corneal angle; cb, ciliary body; co, cornea; co en, corneal endothelium; co ep, corneal epithelium; ir, iris; lens ep, lens epithelium. The photographs are reproduced from reference [23].

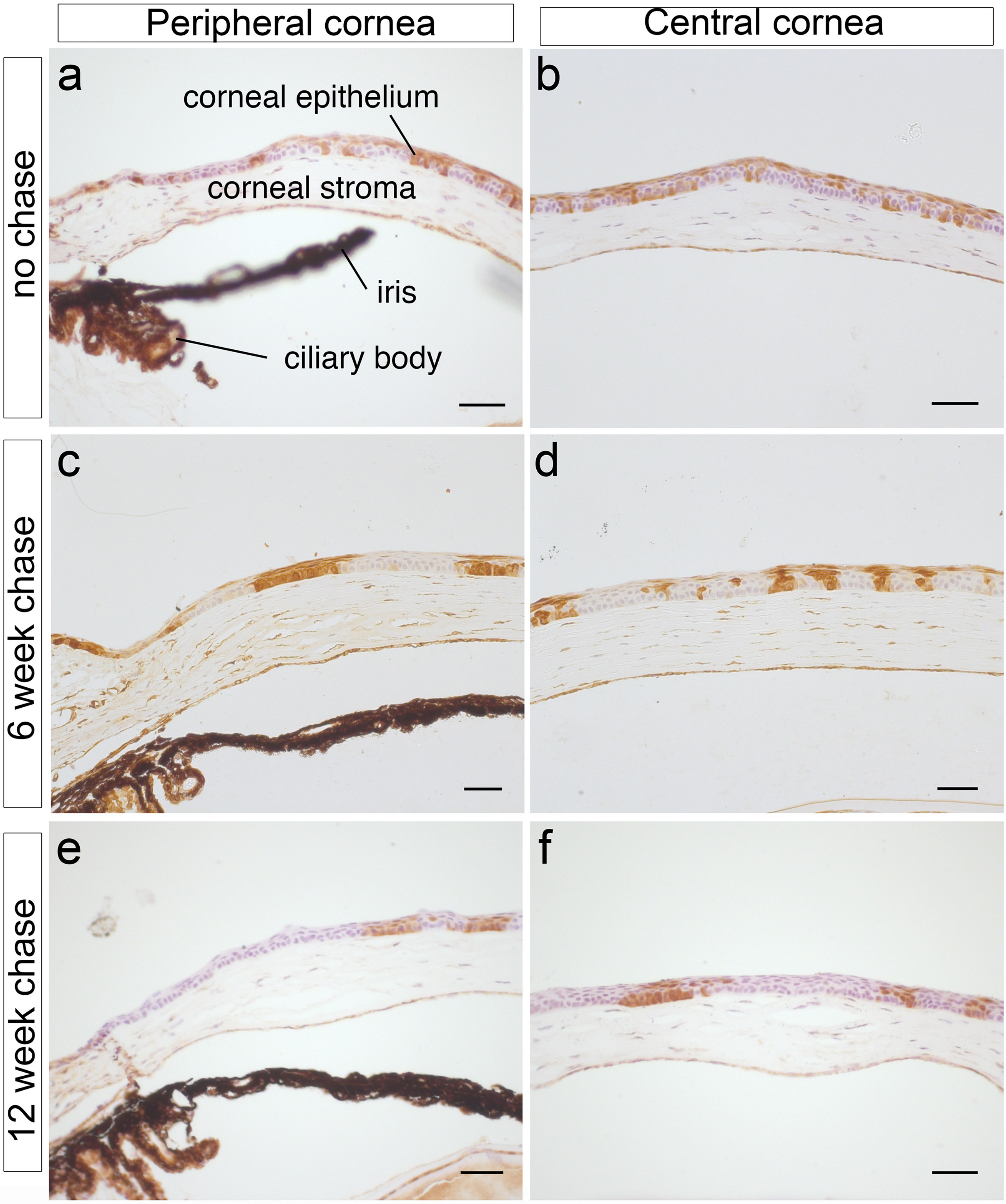

Additional file 2: Fig. S3. Expression of GFP reporter in corneal epithelium of CAG-CreER$^{Tg/-}$;RCE:*loxP* mice after tamoxifen treatment and different chase periods.

Corneal sections from CAG-CreER$^{Tg/-}$;RCE:*loxP*, GFP-reporter mice after treatment with tamoxifen at 12 weeks and different chase periods. Sections were immunostained for GFP (brown DAB endpoint) and counterstained with haematoxylin. GFP staining in the corneal epithelium was patchy in both the peripheral (a, c, e) and central (b, d, f) cornea, three days after tamoxifen treatment (no chase group; a, b) and after chase periods of 6 (c, d) or 12 (e, f) weeks. Scale bars = 50 µm.

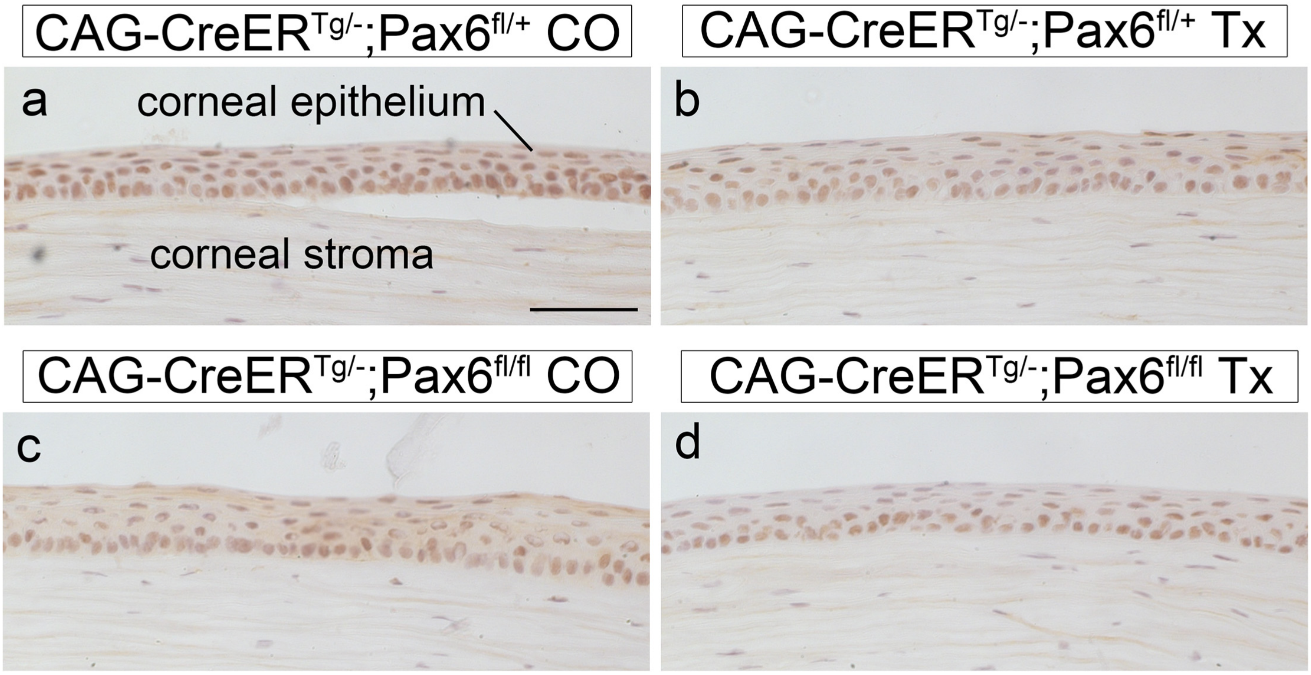

Additional file 2: Fig. S4. Pax6 immunohistochemistry of CAG-CreER$^{Tg/-}$;*Pax6$^{fl/+}$* and CAG-CreER$^{Tg/-}$;*Pax6$^{fl/fl}$* corneal epithelia after a 6-week chase period.

Central region of the adult corneal epithelium, immunostained for Pax6 (brown DAB endpoint) and counterstained with haematoxylin. Samples were from CAG-CreER$^{Tg/-}$;*Pax6$^{fl/+}$* (a, b) and CAG-CreER$^{Tg/-}$;*Pax6$^{fl/fl}$* (c, d) mice, six weeks after treating control mice with corn oil (a, c) and experimental mice with tamoxifen (b, d). Pax6 staining was detected in control and experimental mice from both genotypes. Scale bar = 50 µm. Abbreviations: CO, Corn oil treatment; Tx, Tamoxifen treatment. Mice were on a predominantly CD-1 genetic background and were produced for a study of Pax6-depletion in other adult tissues [18].

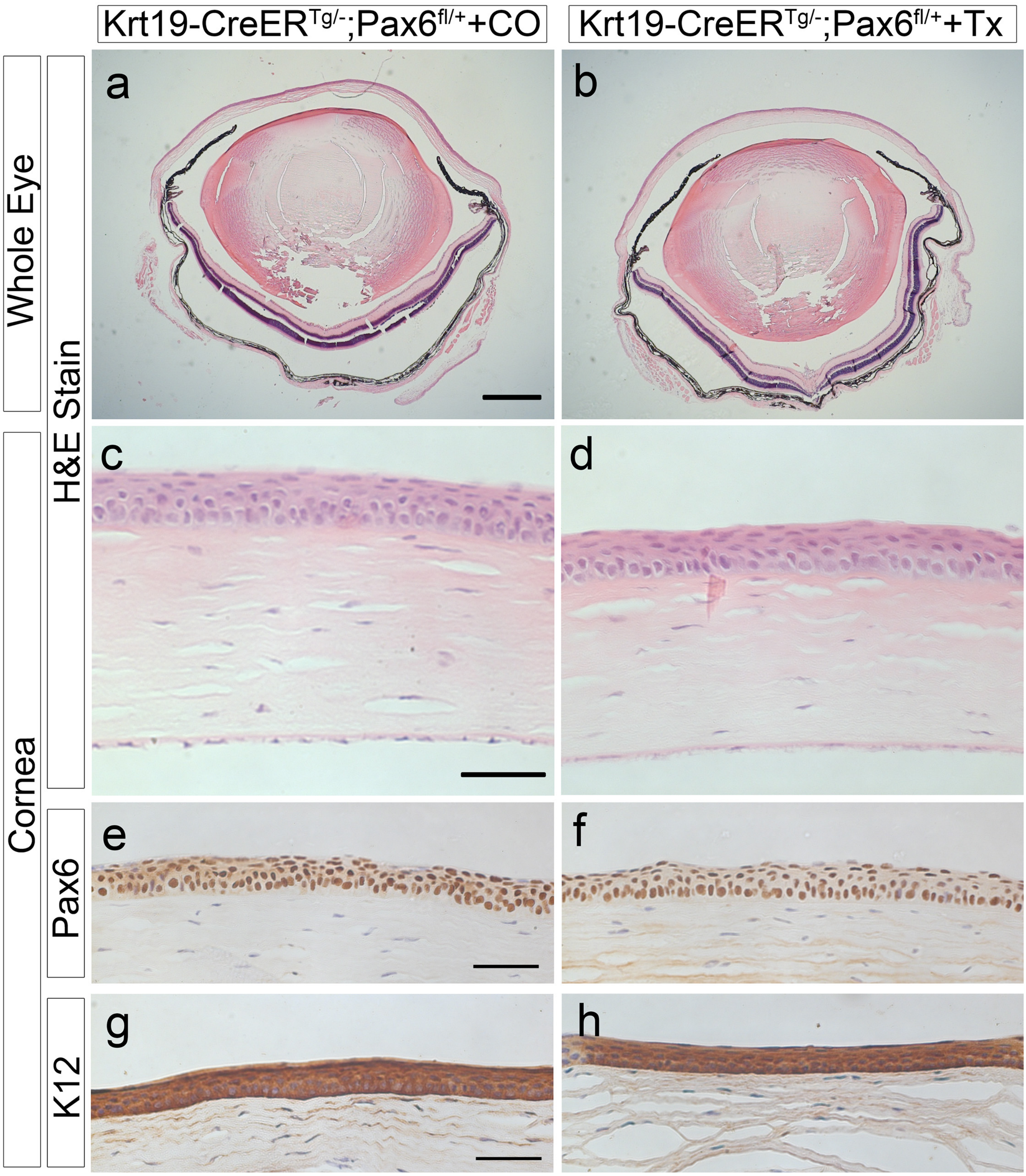

Additional file 2: Fig. S5. Histology and immunohistochemistry of *Krt19*-CreER$^{Tg/-}$;Pa*x6*$^{fl/+}$ tissues after a 12 week chase period.

(a–d) H&E stained sections of whole eyes (a, b) and central cornea (c, d) of corn oil treated controls (a, c) and tamoxifen treated *Krt19*-CreER$^{Tg/-}$; *Pax6*$^{fl/+}$ mice (b, d), 12 weeks after treatment. **(e–h)** Immunostained sections (brown DAB endpoint) for Pax6 (e, f) and keratin 12 (g, h), showing strong staining in both control (e, g) and tamoxifen-treated (f, h) groups. Scale bars: a (for a, b) = 500 µm; c (for c, d), e (for e, f) and g (for g, h) = 50 µm. Abbreviations: CO, corn oil treatment; Tx, tamoxifen treatment.

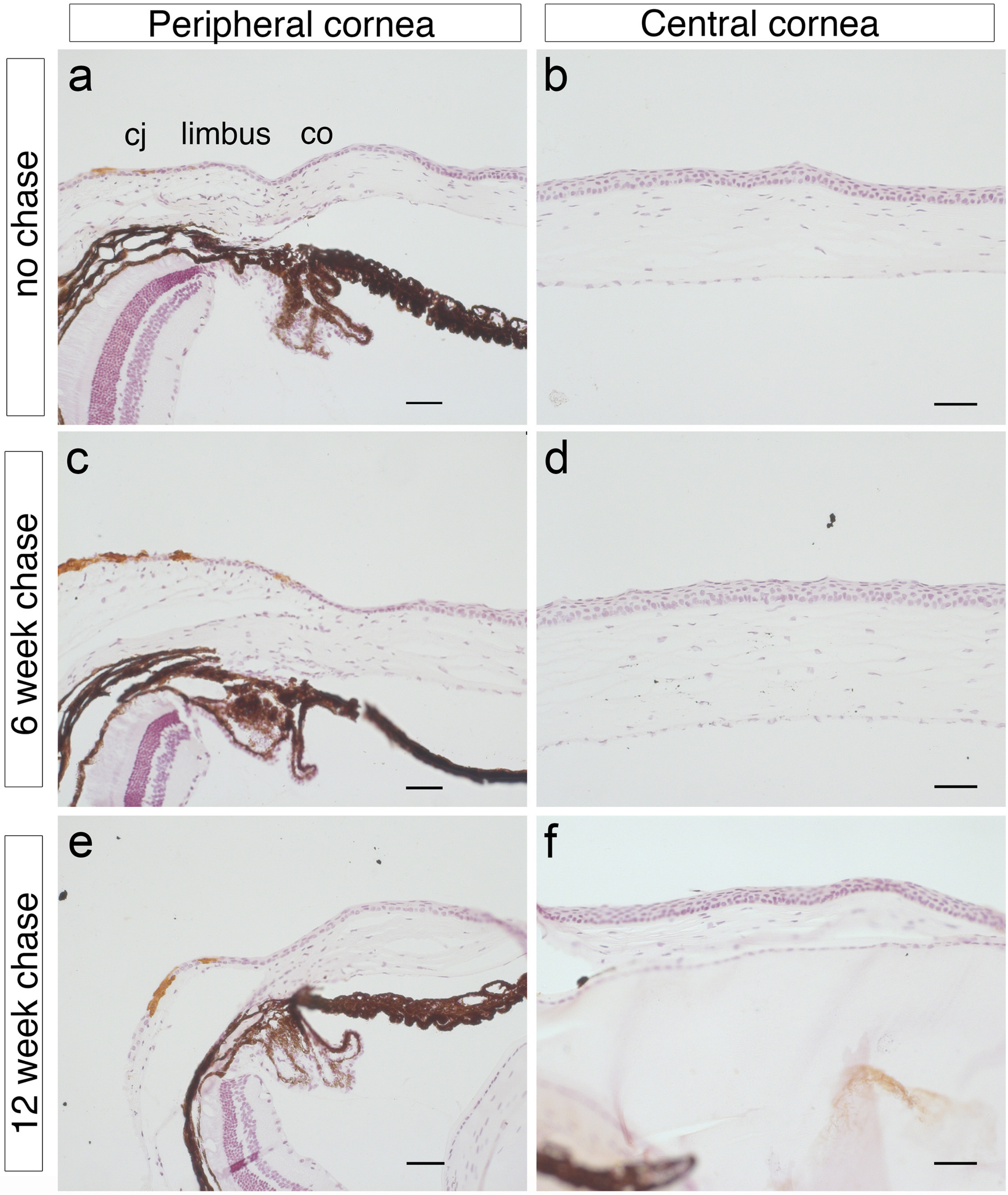

Additional file 2: Fig. S6. Expression of GFP reporter in corneal epithelium of *Krt19*-CreER$^{Tg/-}$;RCE:*loxP* mice after tamoxifen treatment and different chase periods.
Corneal sections from *Krt19*-CreER$^{Tg/-}$;RCE:*loxP*, GFP-reporter mice after treatment with tamoxifen at 12 weeks and different chase periods. Sections were immunostained for GFP (brown DAB endpoint) and counterstained with haematoxylin. GFP staining in the corneal epithelium was patchy in the conjunctiva, sparse in the limbal region and absent from the peripheral cornea (a, c, e) and central cornea (b, d, f), three days after tamoxifen treatment (no chase group; a, b) and after chase periods of 6 (c, d) or 12 (e, f) weeks. Scale bars = 50 µm. Abbreviations: cj, conjunctiva; co, cornea.
